# Age-related processing strategies and go–nogo effects in task-switching: an ERP study

**DOI:** 10.3389/fnhum.2015.00177

**Published:** 2015-04-13

**Authors:** Zsófia A. Gaál, István Czigler

**Affiliations:** Institute of Cognitive Neuroscience and Psychology, Research Centre for Natural Sciences, Hungarian Academy of SciencesBudapest, Hungary

**Keywords:** aging, task-switching paradigm, event-related potentials, go–nogo

## Abstract

We studied cognitive and age-related changes in three task-switching (TS) paradigms: (1) informatively cued TS with go stimuli, (2) informatively cued TS with go and nogo stimuli, (3) non-informatively cued TS with go and nogo stimuli. This design allowed a direct comparison, how informative and non-informative cues influenced preparatory processes, and how nogo stimuli changed the context of the paradigm and cognitive processing in different aging groups. Beside the behavioral measures [reaction time (RT), error rate], event-related potentials (ERPs) were registered to the cue and target stimuli in young (*N* = 39, mean age = 21.6 ± 1.6 years) and older (*N* = 40, mean age = 65.7 ± 3.2 years) adults. The results provide evidence for declining performance in the older group: they had slower RT, less hits, more erroneous responses, higher mixing costs and decreased amplitude of ERP components than the participants of the younger group. In the task without the nogo stimuli young adults kept the previous task-set active that could be seen in shorter RT and larger amplitude of cue-locked late positivity (P3b) in task repeat (TR) trials compared to task switch trials. If both go and nogo stimuli were presented, similar RTs and P3b amplitudes appeared in the TR and TS trials. In the complex task situations older adults did not evolve an appropriate task representation and task preparation, as indicated by the lack of cue-locked P3b, CNV, and target-locked P3b. We conclude that young participants developed explicit representation of task structures, but the presence of nogo stimuli had marked effects on such representation. On the other hand, older people used only implicit control strategy to solve the task, hence the basic difference between the age groups was their strategy of task execution.

## Introduction

The task-switching (TS) paradigm is a suitable method to study working memory (WM) and cognitive flexibility – psychological processes that are affected by cognitive aging. We compare three versions of the paradigm to reveal how preparation and task structure influence the underlying cognitive processes in young and older adults.

The basis of the TS paradigm is alternation between two (or more) simple tasks conveyed by internal or external cues. The task requires maintaining task-sets, and switching between them (task-set refers to the stimulus-response rules that are specific to the given task), as well as preparing and implementing responses. Behavioral studies show that responses of repeat trials are slower in mixed-task blocks (in which two or more task-sets are in operation) compared to single-task blocks (with only one task-set). This difference in reaction time (RT) is the mixing costs (MCs) reflecting higher WM demand on mixed-task. Switching costs (SC) manifest slower responses in switch trials compared to repeat trials in mixed-task blocks and constitute an index of flexibility (see Kiesel et al., 2010 for a review). Main theories propose that at the beginning of the current trial the previous task-set is still active. If a switch occurs, the reconfiguration of the task-set is required (Rogers and Monsell, 1995), or according to another view, extra time is needed for resolving the interference between the previously active and the current task-set (Wylie and Allport, 2000).

In aging studies MC were found to be larger for older adults (e.g., Karayanidis et al., 2010), the added WM load of maintaining and manipulating multiple task sets cause age differences that exceed age-related cognitive slowing (Wasylyshyn et al., 2011). Results concerning SC are ambiguous; some authors reported an increased (Hillman et al., 2006), or a decreased (Kray, 2006), or unchanged (Kopp et al., 2014) SC. The differences in age-related changes of MC and SC also show that the two effects connect to distinct aspects of cognitive control. In fMRI studies activation of separate brain regions were associated with MC and SC (e.g., Braver et al., 2003). While young adults show activation in dorsolateral and medial frontal lobe during TS, older people activate these areas during TS and TR as well, probably to compensate deterioration processes of the aging brain (DiGirolamo et al., 2001).

Models of TS separate several processes operating within cue-target interval and after target onset. E.g., Rubinstein’s stage model (Rubinstein et al., 2001) distinguishes task processes and executive control processes. Task processes are stimulus identification, response selection and movement production. Executive control processes have two distinct stages. Goal shifting keeps the content of declarative WM up-to-date by inserting and deleting task goals. Rule activation has two complementary functions: the activation of the current task-set and the inhibition of the prior one.

The ERP method is convenient to distinguish these hypothesized processes. Its time resolution gives the opportunity to separate phases that happen before the response. Consistent with the idea of goal shifting, a larger early cue-locked positivity was found for informatively cued trials compared to non-informatively cued trials (non-informative cues index only the timing of the upcoming stimulus, but do not inform about the task itself). The amplitude of this component correlated with the activity in the dorsolateral prefrontal cortex, known to be involved in top–down control during goal-directed behavior (Jamadar et al., 2010). A later cue-locked positivity showed higher amplitude in the informatively cued TS trials relative to task repetition (TR) trials (e.g., Kieffaber and Hetrick, 2005; Astle et al., 2006; Nicholson et al., 2006). It correlated with the activity in the posterior parietal cortex, and was connected to category-response rule activation (Jamadar et al., 2010). The amplitude is modulated by both cue and TS (Perianez and Barcelo, 2009). While the early cue-locked ERP is associated with stimulus feature processing, the late posterior positivity is linked to strategic processes (Karayanidis et al., 2010). A negative shift was also found before target stimulus onset; the CNV is related to motor and cognitive preparation for the current task (Walter et al., 1964), and its amplitude is larger in participants showing good performance (Hohnsbein et al., 1998). Astle et al. (2006) found in a TS paradigm with nogo stimuli reduced amplitude in go after nogo trials compared to go after go trials. In their interpretation this negativity reflects conquering inhibition from previous trial. Others found larger amplitude for better prepared trials, indexing anticipatory control and successful preparation (Lavric et al., 2008).

N2 can reflect to cognitive control such as response inhibition, response conflict and error monitoring (Folstein and Van Petten, 2008). Gajewski et al. (2010) argued that the target-locked N2 component reflects response selection – the amplitude increases as response selection becomes more difficult. N2 is also enhanced in TS trials relative to TR trials (e.g., Karayanidis et al., 2003). The P3b component is thought to index WM update processes (Donchin and Coles, 1988) or the closure of a perceptual cycle (Verleger, 1988). Its amplitude is smaller in TS trials than in TR trials (e.g., Karayanidis et al., 2003; Gajewski et al., 2010) which can be a result of effortful processing in TS trials in which there is a greater WM demand, or show a strengthened representation in TR trials. Aging studies usually report reduction in the above mentioned cue- and target-locked components (Gajewski et al., 2010; Karayanidis et al., 2011), suggesting age-related impairment of connected cognitive processes.

We designed the current experiment to examine the aging effects and their changes with a cognitive training on behavioral and neural correlates of cognitive processes occurring during TS paradigms. In this paper we only show the age-related differences observed before the training process, the training effects are summarized elsewhere (Gaál and Czigler, in preparation). Hence the control and experimental groups of the training procedure are merged and we do not discuss the results of those tests and paradigms that were designed to indicate transfer effects.

We introduced three different TS paradigms that are suitable to demonstrate how task complexity influences the underlying processes. The easiest paradigm contained only go stimuli and we could use it as a classical reference task that is similar in its structure to most of the commonly used tasks. In the other paradigms the tasks were more complex as we inserted nogo stimuli in 25% of the trials. This modification allows the observation of the effect of inhibitory control and greater memory demand – processes that are thought to be affected by aging (Hasher and Zacks, 1988). Here we discuss the consequences of high WM demand. Another parameter that influenced task difficulty was the introduction of informative vs. non-informative cues. Informative cues provide an opportunity for anticipatory processes while non-informative cues provide information only about stimulus timing but not about an impending TS or TR, hence the latter is more difficult to execute.

On the basis of previous studies we hypothesized that older adults would show reduced WM capacity and cognitive flexibility manifesting in higher MC and SC, and in a decreased amplitude of the observed ERP components: cue-locked early and late positivity, CNV, target-locked N2 and P3 components. These differences were hypothesized to be modified by task context changes.

## Materials and Methods

### Participants

This study ran with the participation of young (18–25 years) and older (60–75 years) women. Demographic data and test scores are shown in **Table 1**. All participants were right handed, had normal or corrected-to-normal vision, and had no history of any kind of neurological or psychiatric disease.

**Table 1:** **Demographic data and WAIS test scores in young and older groups (mean, SD)**.

	Young	Older	*t*-test			
N	39	40				
Age (years)	21.6 ± 1.6	65.7 ± 3.2				
Activity (hours/week)	5.9 ± 5.4	12.6 ± 8.2	-4.241^∗^			
IQ (WAIS-IV)	108.7 ± 13.3	119.0 ± 16.1	-3.101^∗∗^			

	**Young scaled score**	**Older scaled score**	***t*-test**	**Young raw score**	**Older raw score**	***t*-test**

**Verbal comprehension**	31.8 ± 6.1	38.1 ± 7.0	-4.219^∗∗^			
Similarities	10.8 ± 2.6	12.3 ± 2.0	-2.868^∗∗^	26.5 ± 3.8	26.0 ± 3.6	0.586
Vocabulary	10.5 ± 2.8	13.0 ± 3.6	-3.484^∗∗^	28.1 ± 7.5	34.6 ± 9.8	-3.000^∗∗^
Information	10.5 ± 2.8	12.7 ± 3.1	-3.310^∗∗^	15.5 ± 4.0	18.3 ± 4.3	-3.011^∗∗^
**Perceptual reasoning**	31.5 ± 6.8	31.0 ± 5.9	0.394			
Block Design	10.6 ± 3.2	9.8 ± 2.6	1.161	47.8 ± 11.8	33.0 ± 10.7	5.870^∗∗^
Matrix Reasoning	10.6 ± 1.5	11.0 ± 2.8	-0.707	19.9 ± 2.1	14.0 ± 4.8	7.058^∗∗^
Visual Puzzles	10.1 ± 3.1	10.3 ± 2.5	-0.272	17.3 ± 4.0	11.2 ± 3.5	7.236^∗∗^
**Working memory**	20.0 ± 3.9	22.2 ± 4.7	-2.263^∗^			
Digit span	9.6 ± 2.0	11.7 ± 2.9	-1.388	27.0 ± 4.7	22.2 ± 4.1	4.909^∗∗^
Arithmetic	10.4 ± 2.6	11.7 ± 2.9	-2.123^∗^	15.4 ± 2.7	14.8 ± 3.0	0.836
**Processing speed**	25.3 ± 4.0	27.8 ± 3.6	-2.878^∗∗^			
Symbol search	12.2 ± 2.4	13.5 ± 2.4	-2.423^∗^	39.7 ± 6.8	27.5 ± 5.3	8.991^∗∗^
Coding	13.1 ± 2.8	14.3 ± 1.9	-2.235^∗^	84.4 ± 12.2	59.9 ± 10.5	9.611^∗∗^

*Red boxes indicate better results in older adults, green boxes index better performance in young adults. ^∗^p < 0.05; ^∗∗^p < 0.01.*

The protocol was approved by the Joint Psychological Research Ethics Committee and a written informed consent was obtained from all participants.

### Procedure

WAIS-IV was completed the first time the participants came to the laboratory and they were asked about their health status and habits. EEG was recorded on the second and the last sessions which were conducted 1 month apart. The control groups participated in these three sessions, the training groups accomplished eight 1-h long training sessions as well. In this study we present only data collected during the first and second sessions and show aging effects. The order of the tasks during the EEG sessions was the following: (1) EEG recording with eyes closed and open, (2) informatively cued TS paradigm with nogo stimuli – letter classification and parity task, (3) non-informatively cued TS paradigm with nogo stimuli – letter classification and parity task (near transfer task), (4) informatively cued TS paradigm – color and shape classification (near transfer task), (5) Attentional Network Test (far transfer task).

**FIGURE 1:**
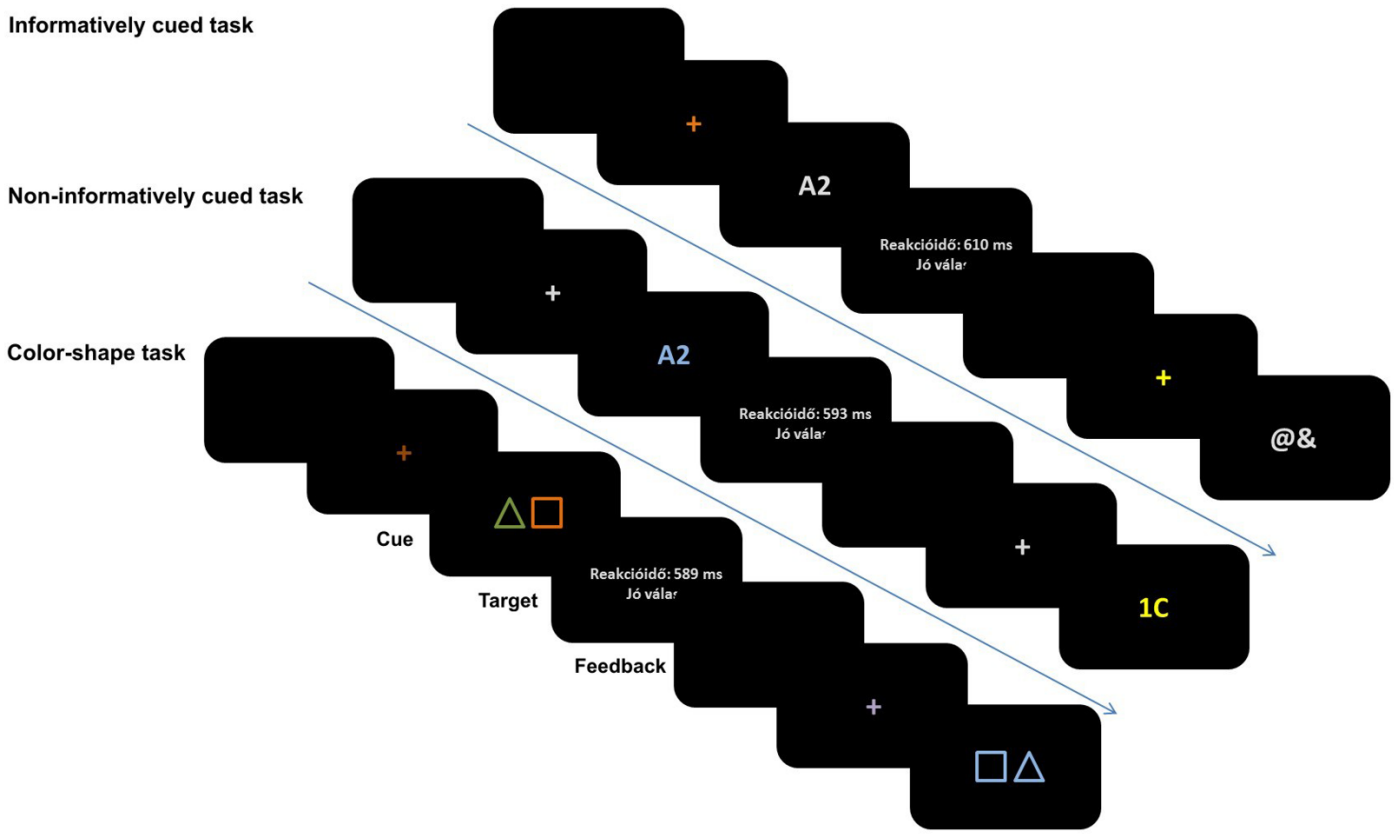
Illustration of the sequence of stimuli in the three types of task-switching paradigms.

**FIGURE 2:**
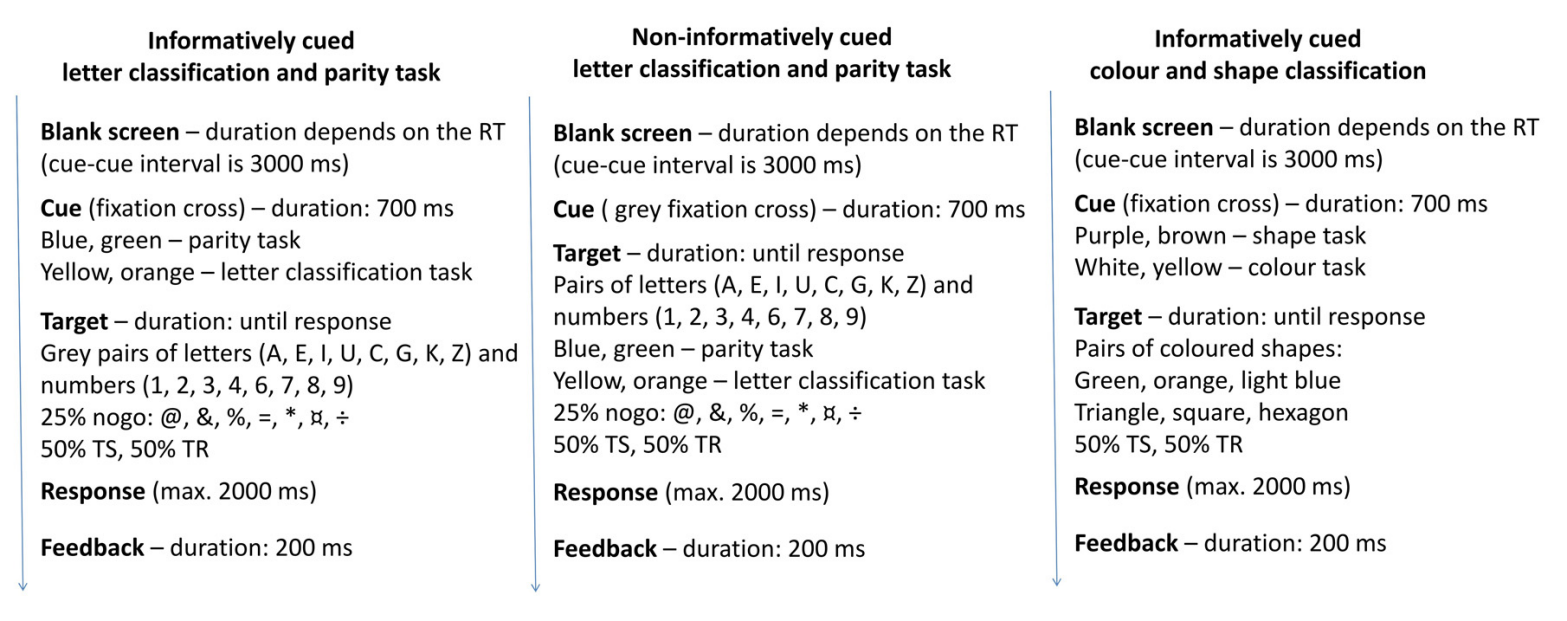
The schemas of the three types of task switching paradigms with the details of stimulus presentation.

Electrophysiological recording was performed in an electrically and acoustically shielded room. Participants were seated in front of a monitor at a distance of 125 cm. Stimuli were presented by Presentation software in the center of the black screen (LG 915FT Plus 19”; 800 × 600 pixels at 60 Hz presentation rate). The TS paradigms are summarized in **Figures 1 and 2**.

Prior to every task participants were given oral and written (on the screen) instructions followed by a practice block.

#### Informatively Cued Task

The first task was an informatively cued TS paradigm with nogo stimuli – letter classification and parity task (informatively cued task). Each trial started with a colored cue for 700 ms which was replaced by a gray target. The target stimuli were removed after response or 2000 ms and a feedback was seen in the screen (Don’t press a button! or the RT in ms and Correct response!/Incorrect response!/Press the button more quickly! – in Hungarian) for 200 ms.

Target stimuli were pairs of gray letters and numbers (1.8^∘^ × 1.4^∘^). The participants had to respond according to the color of the cue. Yellow and orange cues were assigned to letter classification task (vowel/consonant decision), blue and green cues to number classification task (odd/even decision). Cue color was never repeated on successive trials to distinguish TS from cue switching. Participants pressed the button of a gamepad with their left or right index finger and response mapping was counterbalanced across them. The letter and the number of a target were incongruently mapped to response hand but every block included four congruently mapped targets to avoid the strategy of attention to only one of the stimuli (e.g., letters only). Responses for congruently mapped targets and the next trial were excluded from further analysis. In 25% of the trials a special character was shown as a target, which indexed that no button press was needed (nogo trial).

Stimuli were presented in a pseudorandom order with 50% switch probability and with the restriction that (1) two nogo trials could not appear in succession, (2) the same target could not be repeated on successive trials, (3) no more than three TS or TR could follow in succession.

Single tasks were executed in two blocks each for letter classification and parity tasks with 104 stimuli. Mixed tasks were presented in 10 blocks with 580 stimuli altogether.

#### Non-Informatively Cued Task

The second task was a non-informatively cued TS paradigm with nogo stimuli – letter classification and parity task (non-informatively cued task). The order of the non-informatively cued task was the same as seen in the informatively cued paradigm above with only two differences: (1) the color of the cue was gray, (2) the color of the targets were yellow or orange (letter classification task), or blue or green (number classification task).

#### Color-Shape Task

The third task was an informatively cued TS paradigm – color and shape classification (color-shape task). Nogo stimuli were not included in this paradigm. The stimuli were pairs of green, orange and light blue triangles, squares and hexagons (5.5^∘^ × 2.75^∘^). Cue color purple and brown indexed the shape task (identical/different), white and yellow were used for the color task (identical/different). Timing, feedback and the restrictions for the stimuli were the same as seen in the other TS paradigms. Single tasks were executed in one block each for shape and color classification tasks with 48 stimuli. Mixed tasks were presented in five blocks with 240 stimuli altogether.

### ERP Recording

The EEG was recorded by NuAmps amplifiers (bandpass: DC-70 Hz) using NeuroScan 4.4 software (sampling rate: 1000 Hz) by 35 Ag/AgCl electrodes placed on Fp1, AFz, Fp2, F7, F3, Fz, F4, F8, FT9, FC5, FC1, FC2, FC6, FT10, T7, C3, Cz, C4, T8, TP9, CP5, CP1, CP2, CP6, TP10, P7, P3, Pz, P4, P8, PO9, O1, Oz, O2, PO10, referred to the tip of the nose, with FCz as ground. Vertical and horizontal eye movements were recorded by electrodes placed above and below the left eye (VEOG) and in the outer canthi of the eyes (HEOG). The impedance of the electrodes was kept below 10 kΩ.

### ERP Data Analysis

The first trial of each block, error trials, trials of congruently mapped targets and the following trial were excluded from analysis.

The oﬄine EEG processing started with bandpass filtering (FIR, 0.1 – 30 Hz, 48 dB/oct). Segmentation for cue-locked ERPs was performed from -100 ms to 700 ms relative to cue onset, for target-locked ERPs from -100 ms to 1000 ms relative to target onset. After removing linear trends, a baseline correction (pre-stimulus interval) and automatic artifact rejection (±80 μV) were executed (on average 11% of epochs were discarded). Finally, cue- and target-locked averages were created.

Here we discuss only the results of go after go trials during the first registration session, nogo and training effects are going to be summarized in a separate paper.

#### Cue-Locked ERPs

For TS paradigms three cue-locked ERP components were analyzed: (1) early positivity (P2) in the 150–250 ms interval, (2) late positivity (P3b) in the 400–500 ms (for young adults in all conditions and older adults in color-shape task) and 450–550 ms (for older adults TS paradigms with nogo stimuli) time window and (3) early CNV in the 600–700 ms period. The intervals were defined after visual inspection of grand averages. Because of the great individual variability we used mean amplitudes for 100 ms long periods.

#### Target Locked ERPs

N2 and P3 components were analyzed in all paradigms for go after go stimuli. We defined latency windows for every condition in every group separately based on the grand averages and took mean amplitudes of a 100 ms long period around the highest negative- and positive-going peaks. The latency windows were the following: (1) Informatively cued task, young adults: 260–360 ms (N2), 500–600 ms (P3), older adults: 320–420 ms (N2). (2) Non-informatively cued task, young adults: 240–340 ms (N2), 400–500 ms (P3), older adults: 270–370 ms (N2), 450–550 ms (P3). (3) Color-shape task, young adults: 200–300 ms (N2), 440–540 ms (P3), older adults: 260–360 ms (N2).

### Statistical Analysis

Statistical analyses were performed by Statistica 12. Data obtained at the Fz, Cz, Pz, and Oz electrodes were analyzed in this study. *t*-test for independent samples was applied for the comparison of the intelligence of the age groups. Repeated measures of ANOVAs for RTs and ERPs were performed with the following factors: Age group (two levels: young and older), Condition (two levels: informatively and non-informatively cued – not used in color-shape task), TSTR (two levels: TS and TR). When appropriate, Greenhouse-Geisser correction was applied, in which cases *p* reflects the corrected values. *Post hoc* analysis was performed by using the Tukey HSD test.

## Results

### WAIS-IV

Older adults had higher IQ scores than young participants [*t*(77) = -3.101, *p* = 0.003]. According to the scaled scores, older participants performed better in Verbal Comprehension [*t*(77) = -4.219, *p* < 0.001], in WM [*t*(77) = -2.263, *p* = 0.026] and in Processing Speed [*t*(77) = -2.878, *p* = 0.005] compared to young adults. In Perceptual Reasoning there was no notable difference between groups (the means of test results and *t*-scores are shown in **Table 1**). These data demonstrate that older participants had higher IQ scores than their peers while the results of young adults were closer to the mean of their age-group. Nevertheless, in case of no transformation of their performance, raw scores reveal an evidence that young adults outperformed older participants in 6 of 10 subtests.

### Behavioral Data

Behavioral data are summarized in **Table 2**.

**Table 2:** **Hits (%), errors (%) and reaction time (RT, in ms) for switch (TS) and repeat (TR) trials; switching costs (SC, in ms) and mixing costs (MC, in ms) are summarized with their mean and standard deviation for the age groups in the three TS paradigms**.

	Informatively cued letter-parity task		Non-informatively cued letter-parity task		Informatively cued color-shape task	


	Young	Older	Young	Older	Young	Older
**Hits**						
TS	89 ± 7	67 ± 15	87 ± 6	67 ± 15	93 ± 6	73 ± 16
TR	88 ± 6	70 ± 15	88 ± 7	71 ± 13	94 ± 5	77 ± 15

**Errors**						
TS	10 ± 7	29 ± 14	13 ± 6	31 ± 15	7 ± 6	26 ± 15
TR	12 ± 5	27 ± 13	11 ± 6	28 ± 12	5 ± 4	21 ± 13

**RT**						
TS	769 ± 145	1095 ± 171	1016 ± 156	1311 ± 148	611 ± 129	1038 ± 188
TR	762 ± 147	1063 ± 158	997 ± 160	1282 ± 143	575 ± 110	1005 ± 229

**SC**						
	7 ± 53	32 ± 61	19 ± 62	28 ± 78	36 ± 41	33 ± 103

**MC**						
	164 ± 109	309 ± 132	402 ± 109	510 ± 109	89 ± 84	338 ± 192

#### Informatively and Non-Informatively Cued Task

Participants responded more quickly in the informatively cued task compared to the non-informatively cued task [*F*(1,77) = 428.06, *p* < 0.001]. RT was prolonged in older adults [*F*(1,77) = 89.501, *p* < 0.001]. RT was also slower in TS trials compared to TR trials [*F*(1,77) = 14.367, *p* < 0.001]. Although the *Condition × TSTR × Age group* interaction was not significant, *post hoc* tests revealed that this TS-TR difference was present only in the older group both in the informatively (*p* = 0.011) and the non-informatively (*p* = 0.037) cued task.

Higher MC was found in non-informatively compared to informatively cued condition [*F*(1,77) = 298.40, *p* < 0.001] and in the older group compared to young adults [*F*(1,77) = 31.225, *p* < 0.001]. We did not observed age-related differences in SC.

Better performance in the informatively cued task compared to the non-informatively cued task was seen only in TS trials [*Condition × TSTR* interaction for hits: *F*(1,77) = 5.797, *p* = 0.018]. *Age group* main effect showed that the young group had more hits [*F*(1,77) = 79.536, *p* < 0.001] and less errors [*F*(1,77) = 72.579, *p* < 0.001] compared to the older. TS-TR difference was seen only in the non-informatively cued task for the older adults: they had more hits [*TSTR × Age group* interaction: *F*(1,77) = 3.013, *p* = 0.087, *post hoc p* = 0.026] and less erroneous responses [*Condition × TSTR × Age group* interaction: *F*(1,77) = 0.318, *p* = 0.574, *post hoc p* = 0.002] in TR trials.

#### Color-Shape Task

In the color and shape classification condition similar behavioral data were observed to the letter classification and parity task. The young adults also had more hits [*F*(1,77) = 53.993, *p* < 0.001] and less errors [*F*(1,77) = 54.460, *p* < 0.001]. *TSTR × Age group* interactions showed that only older adults performed better in TR trials compared to TS: they had more hits [*F*(1,77) = 6.152, *p* = 0.015] and less errors [*F*(1,77) = 7.227, *p* = 0.009], this kind of difference was not seen in the young group.

Older people had slower responses than young adults [*F*(1,77) = 130.54, *p* < 0.001]. Participants were faster in TR compared to TS trials [F(1,77) = 15.071, *p* < 0.001]. MC was higher in older adults [*t*(77) = -7.407, *p* < 0.001], and we did not observe age-related differences in SC.

### Cue-Locked ERPs

**FIGURE 3:**
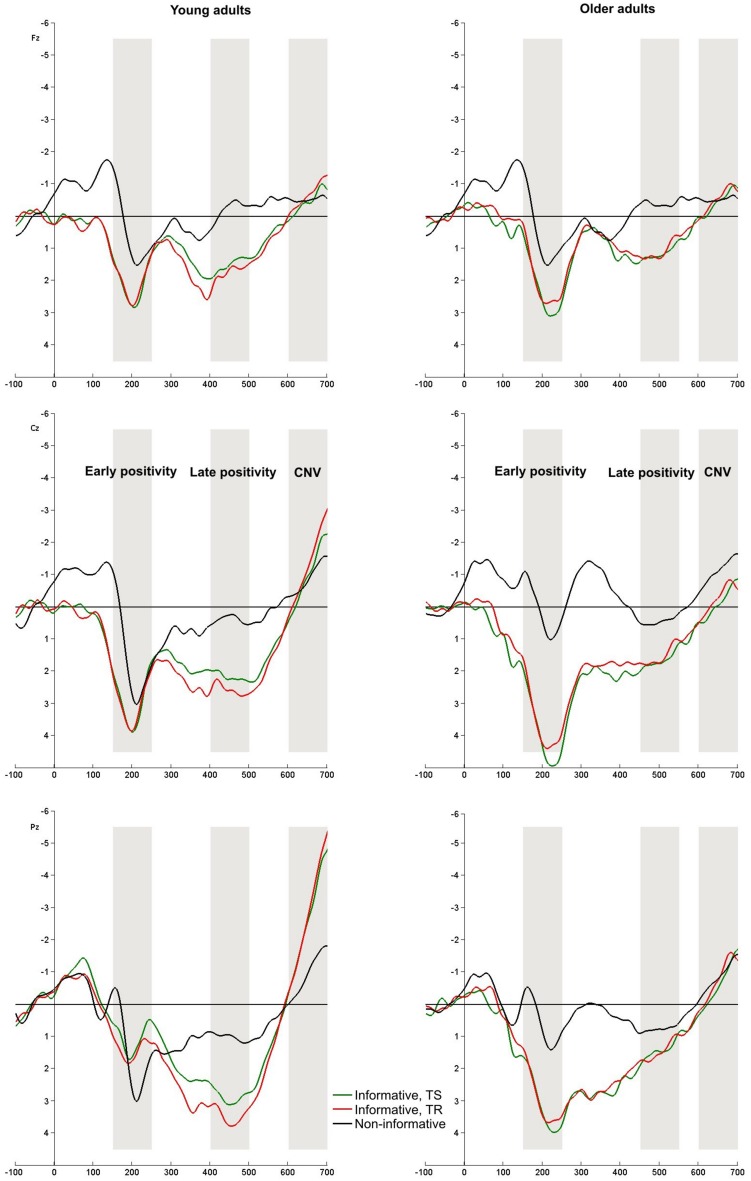
Cue-locked ERPs at Fz, Cz, and Pz electrodes. Informatively cued (informative TS – green, TR – red) and non-informatively cued (black) conditions in young and older groups. Rectangles index the time windows of the analyzed ERPs: early positivity, late positivity, and early CNV.

**FIGURE 4:**
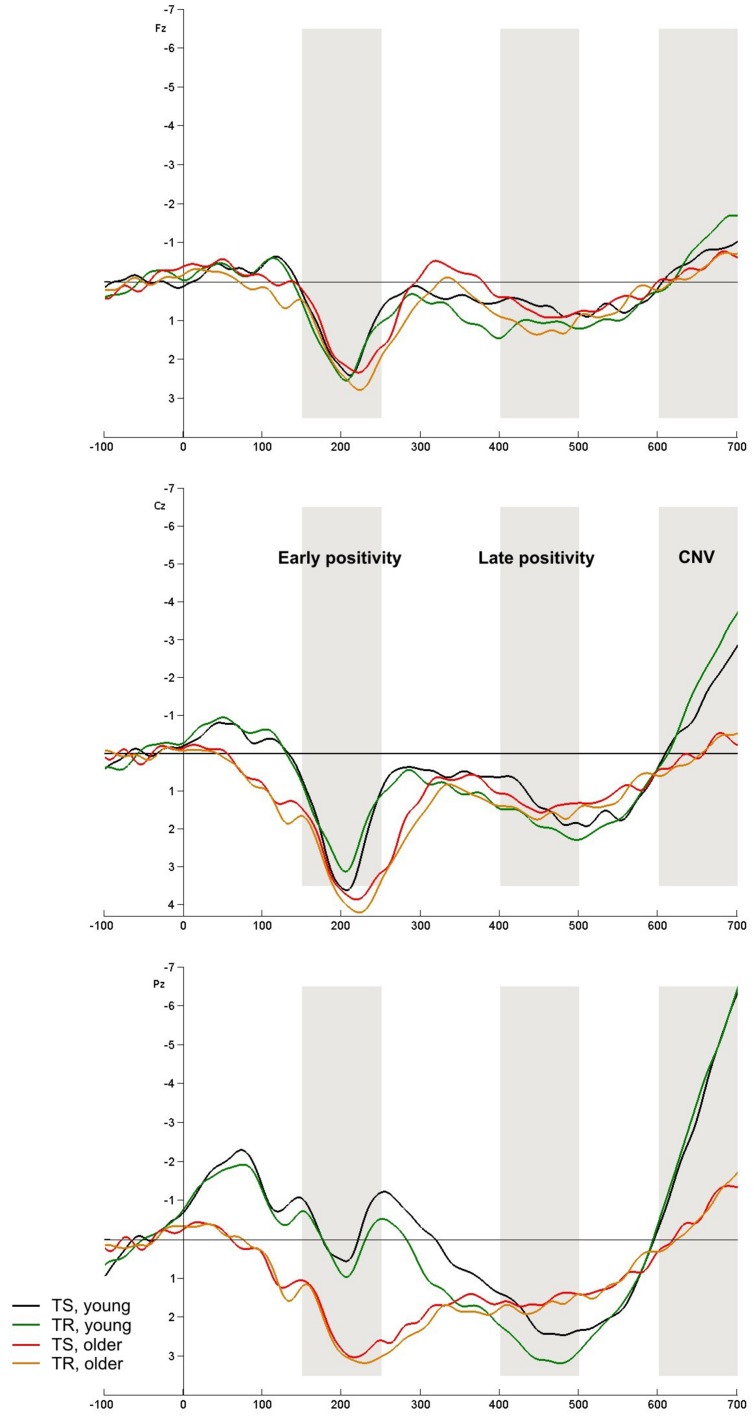
Cue-locked ERPs at Fz, Cz, and Pz electrodes. TS and TR for color and shape classification task in young (TS – black, TR – green) and older (TS – red, TR – orange) groups. The analyzed components are indexed by rectangles: early positivity, late positivity and early CNV.

Cue-locked ERPs are depicted in **Figures 3 and 4**.

#### Early Positivity (P2)

The amplitude of the P2 component did not show *Group* main effect nor TS-TR difference. Higher amplitude was seen in the informatively cued task compared to the non-informatively cued task, but only in older adults [*F*(2,154) = 10.859, *p* < 0.001].

We found *Group* main effect [*F*(1,77) = 8.302, *p* = 0.005] in the color-shape task: the amplitude was higher in the older group compared to young adults.

#### Late Positivity (P3b)

Late positivity was not observed in non-informatively cued task in either groups, and it was not evoked in informatively cued task in the older adults. In young participants the amplitude did not show difference in task switches and TRs. In contrast to these results, the amplitude of P3b potential was reduced in TS trials compared to TR [*F*(1,77) = 10.090, *p* = 0.002] in color-shape task. In this condition age-related differences could not be observed.

#### Early CNV

A negative shift was seen before the target stimulus, which was most enhanced in young adults in the informatively cued conditions [*F*(2,154) = 16.635, *p* < 0.001]. The older adults had smaller amplitude and they did not show difference between informatively and non-informatively cued tasks. The CNV was also seen with higher amplitude in young adults in the color-shape task [*F*(1,77) = 42.208, *p* < 0.001].

### Target-Locked ERPs

**FIGURE 5:**
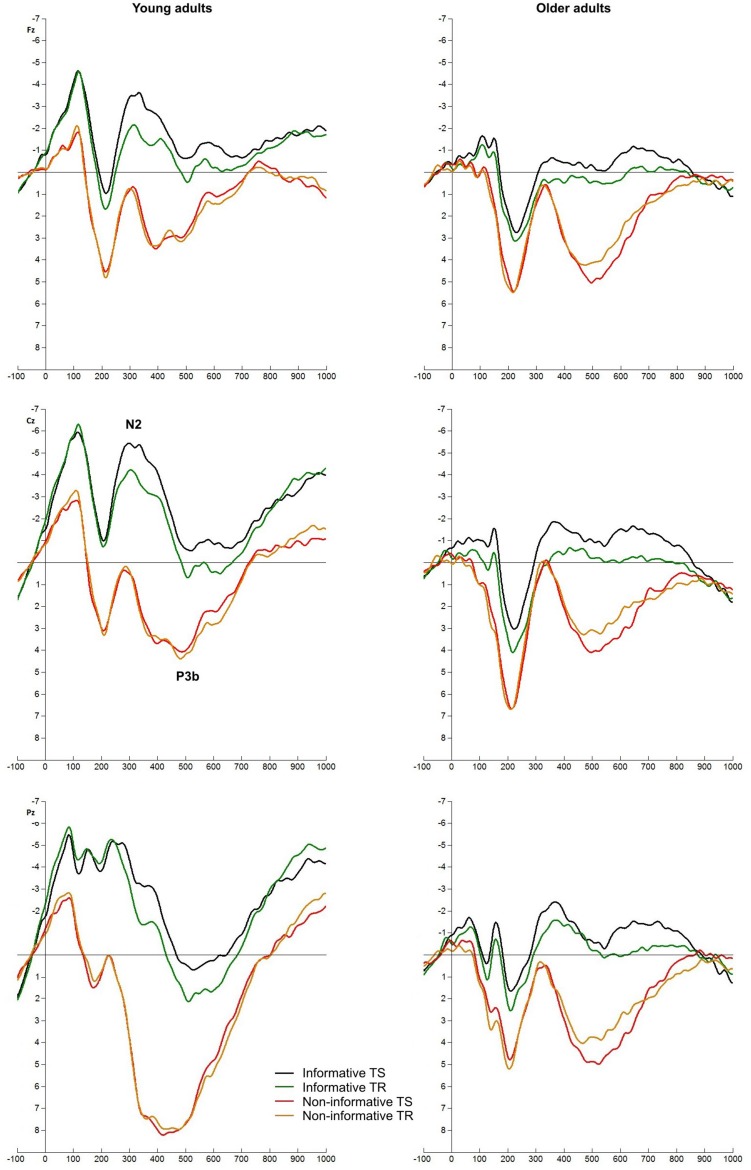
Target-locked ERPs at Fz, Cz, and Pz electrodes in informatively (TS – black, TR – green) and non-informatively cued (TS – red, TR – orange) conditions in young and older groups.

**FIGURE 6:**
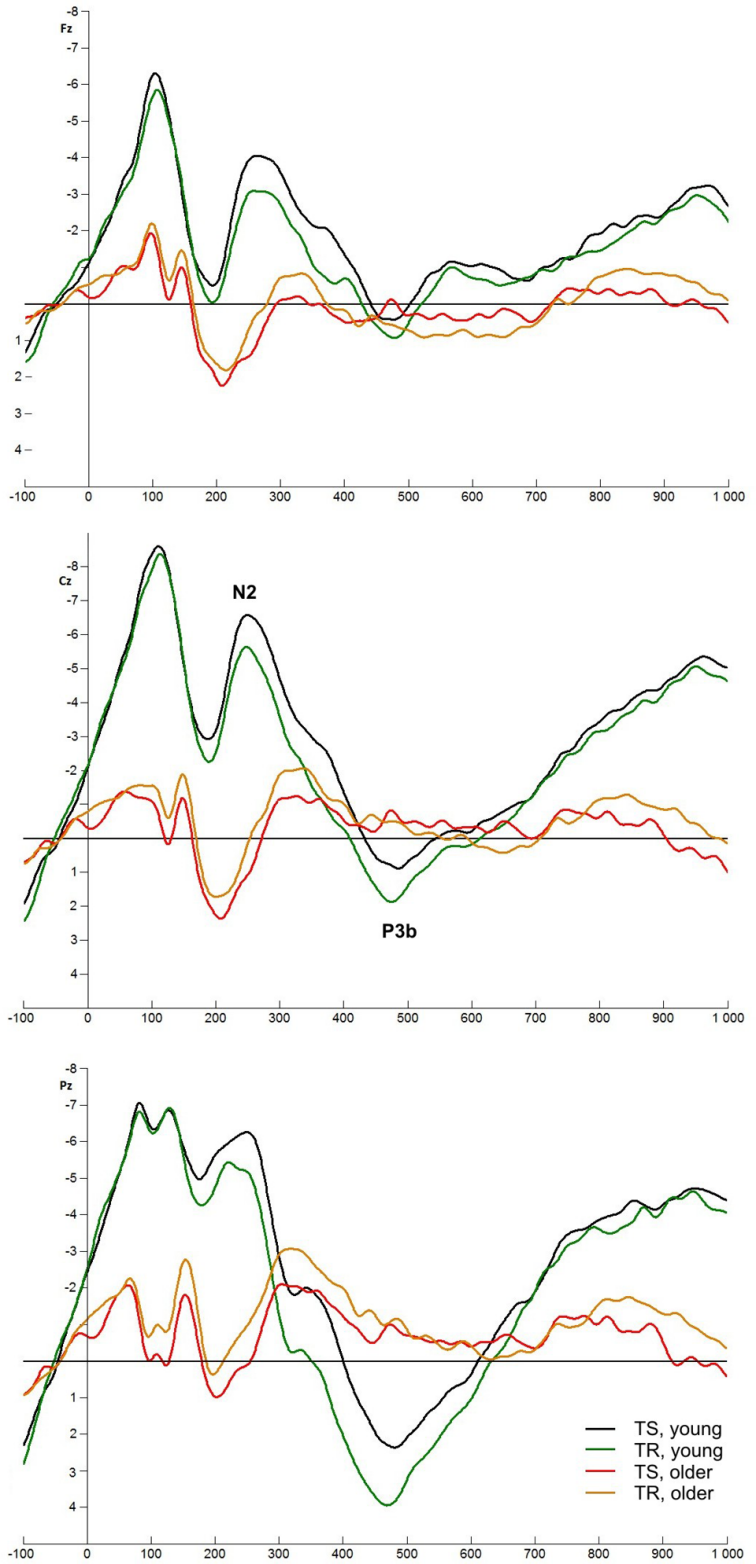
Target-locked ERPs at Fz, Cz, and Pz electrodes. TS (young – black, older – red) and TR (young – green, older – orange) for color and shape classification task.

Target-locked ERPs are shown in **Figures 5 and 6**.

#### N2 Component

N2 amplitude was higher in the informatively cued task compared to the non-informatively cued task [*F*(1,77) = 119.44, *p* < 0.001]. TS-TR difference was found only in the informatively cued task: amplitude was higher in TS compared to TR trials [*F*(1,77) = 10.768, *p* = 0.002]. The age groups did not differ in these tasks. On the contrary, *Age group* main effect was seen in color-shape task: young adults had higher amplitude [*F*(1,77) = 7.308, *p* = 0.008]. The two groups differed in TS trials, but TS-TR difference (higher amplitude in the former condition) was only seen in young participants [*TSTR × Age group* interaction: *F*(1,77) = 12.608, *p* < 0.001].

#### P3b Component

The target stimulus evoked P3b component in young adults in all conditions but this component could not be seen in older people in informatively cued and color-shape conditions. For this reason we analyzed P3b potential only in young adults for these conditions, and compared the groups only in non-informatively cued task.

P3b amplitude was higher in the non-informatively cued task compared to the informatively cued task [*F*(1,38) = 77.736, *p* < 0.001], and enhanced in TR compared to TS in informatively but not in non-informatively cued task [*F*(1,38) = 9.680, *p* = 0.004]. Higher amplitude for TR compared to TS was also seen in the color-shape task [*F*(1,38) = 16.186, *p* < 0.001].

Older participants only had P3b component in non-informatively cued task: their amplitude was here smaller compared to young adults [*F*(1,77) = 6.621, *p* = 0.012].

## Discussion

The aim of the current study was to compare cognitive processing and flexibility in young and older adults across different TS paradigms. Both behavioral and ERP measures revealed that the structure of the task influenced both the stages of cognitive processing and the aging effects. In the easiest version of the TS paradigm we were able to reproduce the main results of the literature, however, in case of nogo stimuli we obtained remarkable alterations, revealing different stimulus processing even in go after go trials. Our study is unique in this regard based on the fact that according to our knowledge no other experiments exist in which informatively and non-informatively cued TS paradigms with nogo stimuli are compared in young and older adults using electrophysiological methods.

Comparing three types of TS paradigms (informatively cued TS with go stimuli, informatively and non-informatively cued TS with go and nogo stimuli) we revealed how nogo stimuli impacted the context of the paradigm (we analyzed only go after go trials in this paper), and how informative and non-informative cues influenced preparatory processes in diverse aging groups.

### Behavioral Changes

Behavioral results showed declining performance in older compared to young adults in all tasks: they had slower RT, less hits and more erroneous responses. MC (the RT difference between repeat trials in mixed- and single-task blocks) was also higher in the older group indicating that they are less effective in maintaining multiple task-sets in WM (Rogers and Monsell, 1995) or as according to Rubin and Merian (2005), it shows the increased interference between task-sets.

In the color-shape task RT was longer in switch trials compared to repeats, as we expected. On the contrary, in TS paradigms with nogo stimuli this difference was only seen in older adults, while no difference could be found among young adults. At the same time we did not find aging effects in SC. These results are in contrast with our expectations, although SC results in older adults are ambiguous (Hillman et al., 2006; Kray, 2006; Kopp et al., 2014). Hillman et al. (2006) found lower SC in physically active older adults compared to their less active peers, while fitness did not affect the performance in young people (Scisco et al., 2008). Based on their self-assessment, older participants involved in our experiment had an active lifestyle; they did 12.6 h activity (exercise, walking, gardening) per week (5.9 h in young participants). The physical activity may have selective benefits for executive functions (Colcombe and Kramer, 2003) which would result in low SC. An alternative explanation to this could be that the two groups used different strategies. The results suggest that older adults kept the previous task-set active as most theories would imply, while young participants used a neutral control strategy in which the activation level of the task-sets are similar (for a review see De Baene and Brass, 2014).

### Cue-Locked Early Positivity

The early parietal positivity with ∼200 ms peak latency is thought to be connected with goal activation or task-set updating (e.g., Eppinger et al., 2007; Jamadar et al., 2010). Jamadar et al. (2010) found that this component emerged in informatively cued trials but not in non-informatively cued trials, when they were mixed in one block. We could not replicate these results in young adults, only older participants showed this difference. Our experiment differed regarding that we had informatively and non-informatively cued trials in separate blocks and we also included nogo stimuli. At the same time we cannot assert that ERP components should be compared in these two conditions. The morphology of the two positive components shows a significant difference. It seems that positivity in the non-informatively cued task is a part of a classical N1-P2 complex, which may reflect recognition and classification of a visual object (Crowley and Colrain, 2004), or protection against interference (Garcia-Larrea et al., 1992), or a preattentive alerting mechanism (Cepoiené et al., 2005). The morphology of the positivity observed in the informatively cued task differed (see **Figure 2**). The amplitude was larger in older participants compared to young adults. If we consider the results of other components and effectiveness of task execution, it may reflect effort, a correlation of task goal activation in WM: young people activated goals corresponding to the given cue, while older participants activated all possible goals.

Over-recruitment of resources in older adults is also in harmony with the results of Berchicci et al. (2012). Using movement-locked ERPs they showed that nogo stimuli affected age group differences. They compared a simple response task with a discriminative response task and found that older participants activated the prefrontal cortex even if only go stimuli were presented, while young adults only activated these areas when the task was more complex, i.e., nogo stimuli were inserted.

### Cue-Locked Late Positivity

The parietal positivity emerging at 400–1000 ms after the cue has several labels such as late parietal positivity (Astle et al., 2006; Jamadar et al., 2010), P3b (Kieffaber and Hetrick, 2005; Goffaux et al., 2006) or differential switch positivity (Karayanidis et al., 2003; Nicholson et al., 2005). However, most of the researchers agree that its amplitude is enhanced in switch trials compared to repeats, and the component is thought to index category-response rule activation. In this regard we did not predict – and the data confirmed our expectation – this component in the non-informatively cued task in which the cue provides only timing information about the task. We found TS-TR difference in the color-shape task only, but the amplitude was larger in repeat trials and not is switch trials. One possible reason for this result is that a negative component precedes the P3b and its amplitude is higher in TS trials compared to TR trials causing smaller P3b amplitude. In the informatively cued letter-parity task the P3b component did not show TS-TR difference in young adults supporting the idea that they apply a neutral control strategy when nogo stimuli are inserted. A striking outcome was visible in older adults; in this age group no P3b emerged. Probably on account of the large number of stimulus-response assignment (four colors each of which coded a task or the possibility of response withholding) older participants could not evolve an appropriate memory trace, and consequently they could not update it.

### Cue-Locked Early CNV

We removed the cue prior to target onset to maximize task preparation, so the letter-number pair did not involve any information about the task. In older participants the CNV – the electrophysiological correlate of motor preparation – was not emerged in either task conditions (**Figures 2 and 3**). As they could not trace consciously the task, they were not able to prepare the execution of the appropriate response. Obviously CNV is expected to be seen only in informatively cued tasks and not in non-informatively cued condition in which the task is unknown in this period. Accordingly, in young adults we did not find a CNV but the negativity emerged in the informative tasks indexing task preparation. Kopp et al. (2014) theory is also worth considering. He claims that young adults use a proactive control and prepare for the task, while older adults use a reactive control and mobilize their resources only in the knowledge of the task. This difference in their strategy can be reflected in differences of the CNV component.

### Target-Locked N2 Component

With the target-locked N2 component we observed age group differences only in the color-shape task; the amplitude was decreased in older adults compared to young adults. We also found differences between switch and repeat trials, but the results were not consistent. In color-shape task the amplitude was enhanced in TS compared to TR in young people. In the informatively cued letter-parity task higher amplitude was detected in TR trials in both groups, while no difference could be found in the non-informatively cued task. This period of the target-locked ERP was influenced by the late CNV. In young adults the N2 sat on this negative shift, while in the non-informatively cued task and in older adults the CNV did not affect the N2 component. To sum up, we cannot draw a considerable conclusion about N2 from our data.

### Target-Locked P3 Component

The most interesting result is that in informatively cued tasks older adults did not have a P3b component. It shows that they did not evolve an explicit model about the task, their uncertainty was not resolved until the feedback. Taking behavioral data – ∼67% hit rate and difference between TS and TR in RT – we conclude that older participants followed the task implicitly but they did not have an appropriate representation about task rules. In young adults the P3b amplitude was smaller in TS trials than in TR trials in informatively cued tasks. This is consistent with other results (Karayanidis et al., 2003; Gajewski et al., 2010) and reflects higher allocation demand of attentional resources (Polich, 2007). While the P3b component was evoked in both groups – with higher amplitude in young adults – in the non-informatively cued task, TS-TR difference could not be demonstrated.

It is important to note, that while young adults had an average IQ, the older group consisted of highly intelligent and physically active people. In spite of this fact their results did not meet the outcomes observed in the young people.

### Limitations

Limitation of the present study is that the task in the simplest TS paradigm (color-shape task) differed from the other TS paradigms (letter-parity task). We could compare them more reliably if the only difference was the presence or absence of nogo stimuli. The reason for this arrangement was in harmony with the main goal of the original experiment. Namely, the color-shape task was designed to study training effects in order to reveal near transfer effects in a task which is similar but not identical to the training task (informatively cued letter-parity task).

Another issue is the high interpersonal variability. With an increased number of participants, we could have separated the groups for comparison along other parameters, such as IQ, activity, effectiveness or presence of a component. The fact that the age groups were un-matched in their IQ and activity means also a limitation for current study. A methodological issue is that the IQ is a norm-referenced test, the same performance in young and old adults results in different IQ scores. Consequently if we select IQ-matched age-groups, older adults will have decreased performance by definition. At the same time it is more striking that aging effects are also observable in active older people with high IQ compared to less active young adults with average IQ.

### Summary

We studied the changes caused by structural differences in TS paradigms. ERP components proved to be sensitive parameters to index underlying cognitive processes. We found that young adults accomplished goal shifting and rule activation, they prepared and reacted to the task. Task configuration determined their strategy; they kept the previous task-set active in the easier version of the task, however, they changed to a neutral control strategy when nogo stimuli were inserted. For older adults the tasks were highly complex. It seems that they tried to load all possible goals, but given the interference and lack of appropriate model, they were not able to prepare for their response and they used an implicit control strategy to solve the task.

To conclude, cognitive processing strongly depends on the structure of the task and therefore the source of age-related differences varies across tasks.

## Conflict of Interest Statement

The authors declare that the research was conducted in the absence of any commercial or financial relationships that could be construed as a potential conflict of interest.
